# Supplementary material for: Predicting obesity reduction after implementing warning labels in Mexico: A modeling study
Source: PLoS Med. 2020 Jul 28;17(7):e1003221. doi: 10.1371/journal.pmed.1003221 (PMC7386611; doi:10.1371/journal.pmed.1003221)
Supplement: S1 Appendix — (PDF) [file pmed.1003221.s002.pdf]

S1 Appendix for "Predicting obesity reduction after implementing warning labels in Mexico: a modeling study"

Ana Basto-Abreu, PhD[1], Rossana Torres-Alvarez, BS[1], Francisco Reyes-Sánchez, BS[1], Romina González-Morales, MS[1], Francisco Canto-Osorio, MS[1], M. Arantxa Colchero, PhD[2], Simon Barquera, PhD[3], Juan A. Rivera, PhD[4], and Tonatiuh Barrientos-Gutierrez, PhD *[1]

[1] *Center for Population Health Research, National Institute of Public Health,Cuernavaca, Mexico.*
[2] *Center for Health Systems Research, National Institute of Public Health,Cuernavaca, Mexico.*
[3] *Center for Nutrition and Health Research, National Institute of Public Health, Cuernavaca, Mexico.*
[4] *National Institute of Public Health, Cuernavaca, Mexico.*

*Contact information: Center for Population Health Research, National Institute of Public Health, Avenida Universidad 655, Santa María Ahuacatitlán, 62100 Cuernavaca, Morelos, México, [tbarrientos@insp.mx], (52)5554871015.

Contents

1 Baseline consumption of beverages and snacks **3**

2 Caloric change parameters **5**
2.1 Main scenario 5
2.2 Sensitivity analysis 8
2.2.1 Chilean law 8
2.2.2 Mexican law 10
2.2.3 Uruguayan study 12

3 Caloric, weight and BMI changes **13**
3.1 Caloric change 13
3.2 Weight change 13
3.3 Change in BMI 14
3.4 Change in obesity prevalence 14

4 Obesity cases averted **15**
4.1 Obesity cases 15
4.2 Obesity cases averted 15

5 Obesity health costs **16**
5.1 Obesity direct and indirect health costs 16
5.2 Obesity health costs averted 17

6 Complementary results **18**

7 Assumptions and sources of information to estimate the expected impact of warning labels **19**

References **21**

1 Baseline consumption of beverages and snacks

To estimate baseline consumption from beverages and snacks we used data from the semiquantitative food frequency questionnaire (SFFQ) included in the National Health and Nutrition Survey (ENSANUT) from 2016. The datasets are available in ENSANUT website [1]. Table A shows beverages and snacks selected for the main analysis following the list presented by and experimental study on Acton, et al.[2].

Table A: Beverages and snacks selected from ENSANUT's 2016 SFFQ based on the experimental study in Canada.

Type	Food group	Description
Beverages	Industrialized carbonated beverages	Soft drinks (cola and other flavors).
	Industrialized non-carbonated beverages	Flavored waters, nectars, sweetened dairy beverages.
	All milks	Flavored, lactose-free, whole, soy, skim and evaporated milks.
Snacks	Salty snacks	Peanuts, popcorn, pips, broad beans, crackers.
	Cereal-based sweets	Industrialized cookies, pastries, cereal bars.
	Non-cereal based sweets	Chocolate, flan, ice-cream, candies, jellies.
	Solid yogurt	Natural and flavored yogurt.
	Processed meats	Ham, bacon, sausages, chorizo.
	Grains and starchy vegetables	Cornflakes, corn kernels, bread crumbs, yam.
	Fruits	Fresh and dried fruits.
	Vegetables	Fresh, frozen and packaged vegetables.

Table B shows beverages selected for the sensitivity analysis. The selection of beverages was based on the Chilean Law of Food Labeling and Advertising from 2015 to 2017 (see Section 2.2.1 for more details) [3].

Table B: Beverages selected from ENSANUT's 2016 SFFQ based on the Chilean law.

Beverages	High in	Not high in
Soda	Carbonated with added sugar.	Carbonated, no added sugar or total sugar below [6 g/100 mL]
Fruit drinks	Industrialized fruit-flavored drinks with added sugar	Industrialized fruit-flavored drinks with no added sugar or total sugar below [6 g/100 mL]
Dairy products	Flavored milk powders, flavored ready-to-drink milk and flavored dairy.	Plain milk, plain milk powders, and plain dairy substitutes
Waters	Flavored powders and ready-to-drink waters with added sugar.	Flavored powders, no added sugar or total sugar below; mineral water, sparkling water [6 g/100 mL]
Coffee and tea		Instant coffee, roasted coffee
100% fruit and vegetable juices		100% fruit and vegetable juices

Using the variables folio and intp, which identify respectively the household and the individual inside the household, we calculated the baseline consumption of energy (kcal/day) and sodium (mg/day) intake of beverages and snacks for each individual. Using the variables that identify uniquely the individual, we merged the three data-sets (chronic diseases, household and food frequency questionnaires). The complete process was performed using R statistical software [4]. The resulting data-set contains the variables detailed in Table C and new variables estimated in the following sections.

Table C: Variables in final data-set.

Name	Variable
intp	Identifier for each individual in ENSANUT's dataset.
folio_c	Unique identifier for each household in ENSANUT's dataset (to merge with nutritional databases).
folio	Unique identifier for each household in ENSANUT's dataset (to merge with anthropometric databases).
age_cat	Age category. Levels: "20 to 39", "40 to 59".
code_upm	Identifier of primary sampling unit.
pondef	Complex survey weight.
est_var	Strata for the estimation of variances accounting for survey design.
sex	Sex of the individual, coded as 1: "male"; 2: "female".
age	Age (yrs).
weight_baseline	Weight at baseline (kg).
height	Height (cm).
bmi	Body mass index before intervention (kg/m^2).
bmi_cat	Categories of "obesity" (1), "else" (0) at baseline.
ses	Socioeconomic level, divided in tertiles, using the weighted sample.
kcal_tot_snacks_bev	Daily total caloric intake from snacks and beverages at baseline (kcal).
sodium_tot_snacks_bev	Daily total sodium intake from snacks and beverages at baseline (mg).
bev_ml_tot	Beverages consumption at baseline (ml).
bev_kcal_tot	Beverages consumption at baseline (kcal).
bev_sodium_tot	Sodium consumption from beverages at baseline (mg).
snack_gr_tot	Snacks consumption at baseline (gr).
snack_kcal_tot	Snacks consumption at baseline (kcal).
snack_sodium_tot	Sodium consumption from snacks at baseline (mg).
bev_kcal_red	Change in energy consumption from beverages after intervention (kcal).
bev_sodium_red	Change in sodium consumption from beverages after intervention (kcal).
snacks_kcal_red	Change in energy consumption from beverages after intervention (kcal).
snacks_sodium_red	Change in sodium consumption from beverages after intervention (kcal).
kcal_red_tot	Total change in energy after intervention (kcal).
sodium_red_tot	Total change in sodium after intervention (kcal).
final_weight	Weight after intervention (kg).
change_weight	Change in weight after years (kg).
bmifinal	Body mass index after intervention (kg/m^2).
change_bmi	Change in body mass index after 5 years intervention (kg/m^2).
change_bmicat	Categories of "obesity" (1), "else" (0) after intervention.

2 Caloric change parameters

We conducted a literature review of recent studies that estimated the potential caloric reduction of warning labels. The selected articles had to report caloric reductions from both beverages and solid foods, preferably. Other inclusion criteria were studies with similar warning labelling definitions that reported the single impact of the intervention.

2.1 Main scenario

For the main analysis, the effect parameters came from an experimental marketplace study based in Canada [2]. This study took place in three cities in Canada with 1440 individuals (726 in the control condition and 714 with "high in" experiment) aged 13 years and older. The "high in" warning label in Canada (Fig. A) uses nutrient thresholds to determine whether or not a food would be required to carry the symbol [5]. Table D shows the proposed thresholds. In the experiment, participants were provided with a sum

of money, and presented with multiple products available for purchase with or without a “high in” label condition. This experiment replicated a realistic purchase because participants spent real money and incurred a financial cost for their purchases. Results showed that participants assigned to the ‘high in” conditions purchased less sodium and fewer calories compared to the no label control condition. We selected this experimental study because it was the only one to present results for both beverages and snacks, presented a wider range of participant’s ages and locations and presented a bigger sample (n = 1440).

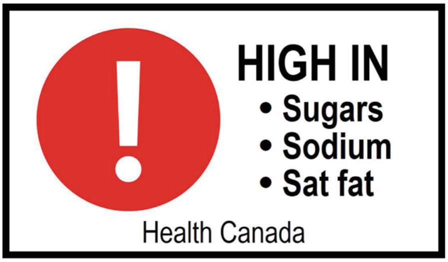

Fig. A: Mandatory warning label in Canada

Table D: Proposed nutrient thresholds for ”high in” warning label in Canada.

Nutrient	Prepackaged foods (15% of the DV)	Prepackaged meals (30% of the DV)
High in Sodium	345 milligrams or more per reference amount and per serving of stated size.*	690 milligrams or more per reference amount and per serving of stated size.*
High in saturated	3 grams or more per reference amount and per serving of stated size.*	6 grams or more per reference amount and per serving of stated size.*
High in sugars	3 grams or more per reference amount and per serving of stated size.*	6 grams or more per reference amount and per serving of stated size.*

*Reference amounts and serving sizes are set out in the Food and Drug Regulations [6].

Table E presents the caloric and sodium reduction including individuals aged 13 and older and including only adults (20 years and older) according to Acton, et al.,2019. Results including only adults were obtained by contacting the first author. The expected caloric change in Acton et al. was higher among 13 years and older than among 20 years and older. To be conservative and because we are estimating the future impact on adults, in this study we used the caloric changes among adults.

Table E: Relative change in calories and sodium after "high in" experiment in Canada by adults and adolescents 13-18 years old.

	**Mean calories purchased (kcal)**		**Mean sodium purchased (mg)**	
	Beverages (liquids)	Foods (solids)	Beverages (liquids)	Foods (solids)
**> 18 years**				
No label (n=608)	102.03	156.28	69.24	154.64
'High in' (n=605)	91.28	151.6	65.4	144.85
Relative change (%)	10.5%	3.0%	5.5%	6.3%
**≤ 18 years**				
No label (n=118)	127.85	191.77	76.11	197.19
'High in' (n=109)	106.49	161.28	62.82	166.41
Relative change (%)	16.7%	15.9%	17.5%	15.6%

Data provided by the main author [2]

2.2 Sensitivity analysis

We based our alternative scenarios in parameters found in countries that previously implemented the warning labels.

2.2.1 Chilean law

In June 2016, Chile implemented the Law of Food Labeling and Advertising, which included a mandatory front-of-package warning labels for sugar sweetened beverages and energy-dense, nonessential foods. Implementation aspects of the Chilean Law of Food Labelling and Advertising can be found elsewhere [7]. Table F presents the three stages of cut-offs for defining regulated foods and beverages.

Table F: Nutrient thresholds and implementation dates of the Chilean Labeling and Advertising Law.

	**26 June 2016**	**26 June 2018**	**26 June 2019**
**Solid food**			
Energy (kcal/100g)	350	300	275
Sodium (mg/100g)	800	500	400
Total sugars (g/100g)	22.5	15	10
Saturated fats (g/100g)	6	5	4
**Liquids**			
Energy (kcal/100ml)	100	80	70
Sodium (mg/100ml)	100	100	100
Total sugars (g/100ml)	6	5	5
Saturated fats (g/100ml)	3	3	3

For this scenario, we considered Table B and the first stage from Table F based on the observational results from Taillie et al. [3]. This study evaluated the changes in purchases of beverages high in sugar, saturated fat, sodium, or calories using national data on household food purchases from before and after the policy implementation (Table G).

Table G: Relative differences of beverages purchases before and after the Chilean Labeling and Advertising Law

	**Relative difference of calories % (95% CI)**
High in	-27.5 (-27.6, -27.5)
Not high in	10.8 (10.8, 10.8)
Total	-7.5 (-7.6, 7.5)

2.2.2 Mexican law

In October 2019, Mexico approved the modification of the law NOM-051 on labeling for nonalcoholic beverages and packaged food [8]. Table H describes the nutrient profiling used for defining prepackaged products targeted in the regulation, and a brief description of the warning label components. Table I presents the nutrient thresholds for food and beverages proposed in the law.

Table H: Implementation aspects of the Mexican Law of Food Labelling

NOM-051-SCFI/SSA1-2010
General labeling specifications for prepackaged food and non-alcoholic beverages-Commercial and health information.

Supplemental nutritional information should be included on the label of prepackaged product that:<br>a) contain additives: free sugars, fats or sodium.<br>b) the energy value, the amount of free sugars, saturated fat, trans fat and sodium meet with the established nutritional profiles.	For solid foods per 100 grams: > 275 kcal total, > 10% of the total energy from free sugars, > 10% of the total energy from saturated fats, > 1% of the total from trans fat; for sodium> 350 mg/100g.<br>For liquids for every 100 milliliters:> 70 kcal total or >10 kcal of free sugars, > 10% of the total energy from free sugars, > 10% of the total energy from saturated fats, > 1% of the total from trans fat; for sodium> 45 mg (non-calorie drinks).
Definition of prepackaged product	Prepackaged product with added free sugars those to which free sugars have been added during the manufacturing process, and ingredients that contain added free sugars.<br>Added prepackaged products of fats, those to which vegetable or animal fats, partially hydrogenated vegetable oils or products and ingredients containing them have been added during the manufacturing process.<br>Added prepackaged sodium product, those to which any salt containing sodium or any ingredient containing added sodium salts has been used as an ingredient or additive during the manufacturing process.
Characteristics of the labels	Octagon black color that contains the legend, white margin on the contour of the octagon, white box background, legend, signature of the Ministry of Health (Figure XX).<br>The stamp(s) should be placed in the upper right corner of the main display surface.<br>The order of inclusion must be from left to right as follows: excess calories, excess sugars, excess saturated fat, excess trans fat, excess sodium. (Figure XX)

Additional legends.	If the ingredient list includes sweeteners, the front precautionary legend should be placed in capital letters "CONTAINS SWEETENERS, NOT RECOMMENDED IN CHILDREN".<br>When the prepackaged product contains added caffeine within the list of ingredients in any quantity, the precautionary legend in capital letters should be included "CONTAINS CAFFEINE AVOID IN CHILDREN"

Table I: Nutrient thresholds and implementation stages of the Mexican Law of Food Labelling.

	**2020-2023**	**2023-2025**
**Solids**		
Energy per 100 grams of solid product	$\geq$ 275 total kcal	$\geq$ 275 total kcal
Sodium per 100 grams of solid product	$\geq$ 350 mg	$\geq$ 1 mg sodium per kcal or $>$ 300 mg
% of total energy from free sugars	$\geq$ 10%	$\geq$ 10%
% of total energy from saturated fat	$\geq$ 10%	$\geq$ 10%
% of total energy from trans fat	$\geq$ 1%	$\geq$ 1%
**Liquids**		
Energy per 100 mL of liquid product	$\geq$ 70 total kcal or $\geq$ 10 free sugar calories	$\geq$ 70 total kcal or $\geq$ 8 free sugar calories
Sodium per 100 mL of liquid product	$\geq$ 45 mg	$\geq$ 45 mg
% of total energy from free sugars	$\geq$ 10%	$\geq$ 10%
% of total energy from saturated fat	$\geq$ 10%	$\geq$ 10%
% of total energy from trans fat	$\geq$ 1%	$\geq$ 1%

For this scenario, we considered the selected beverages from Table B, the first stage from the implementation of the Mexican Law of Food Labelling, and the observational results from Taillie et al. (Table G).

2.2.3 Uruguayan study

An experimental study by Machín et al. evaluated the influence of nutritional warnings on consumers' choice of a snacks [9]. The study included 199 participants from a University whom were asked to complete a task and rewarded with the option to pick a snack from a shelf with 15 products. The control group selected products with no front-of-package nutrition labelling, and the experimental group chose products that featured nutritional warnings as

front-of-package nutrition labelling. Table J presents the reduction in the average calorie, added and sodium content of the selected products caused by nutritional warnings labels.

Table J: Reduction in the average content of calories and sodium of the chosen products from the Uruguayan experimental study.

	**Relative difference %**
Caloric content (kcal)	-11.7
Sodium (mg)	-50.2

For this scenario, we considered the snacks classification from Table A.

3 Caloric, weight and BMI changes

3.1 Caloric change

For each individual k in the sample, we calculated the caloric change from beverages ($\Delta EI_k^{Beverages}$) and snacks (ΔEI_k^{Snacks}) after the warning label implementation as follows:

$$\Delta EI_k^{Beverages} = -reduction \times EI_k^{Beverage}, \tag{1}$$

$$\Delta EI_k^{Snacks} = -reduction \times EI_k^{Snacks}, \tag{2}$$

where $EI_k^{Beverage}$ and EI_k^{Snacks} represent the baseline consumption (in calories) from beverages and snacks, respectively and *reduction* corresponds to each caloric change parameter in Section 2. As a result from Equations 1 and 2, the total energy intake change (ΔTEI_k) was calculated as:

$$\Delta TEI_k = \Delta EI_k^{Beverages} + \Delta EI_k^{Snacks}, \tag{3}$$

By analogy, the total change in sodium intake (ΔNa_k) in milligrams, was calculated using the following equations:

$$\Delta Na_k^{Beverages} = -reduction \times Na_k^{Beverages}, \tag{4}$$

$$\Delta Na_k^{Snacks} = -reduction \times Na_k^{Snacks}, \tag{5}$$

$$\Delta TNa_k = \Delta Na_k^{Beverages} + \Delta Na_k^{Snacks}, \tag{6}$$

where *reduction* corresponds to the sodium change parameters if available.

3.2 Weight change

Body weight change was estimated using the Adult weight change model from Hall et al. [10, 11, 12], which takes into account sex, age, height, initial body weight (BW) and daily changes in energy and sodium. The model was completely programmed in the *bw* package in *R*. A more detailed description of its implementation can be found elsewhere [13].

Considering changes in caloric and sodium intake after a hypothetical warning labeling implementation (Equations 3 and 6), we estimated body weight change ($BW_k(t)$) as follows:

$$BW_k(t) = BW_k^{\text{model}}\big(t + age_k;\ \text{Sex}_k, \text{Height}_k, \text{BW}_k(0), \Delta TEI_k, \Delta Na_k\big), \tag{7}$$

where t stands for the number of days after the intervention, k for each individual on the sample, $\text{BW}_k(0)$ the initial body weight and ΔTEI_k, ΔTNa_k the total caloric and sodium changes, respectively .

As shown in Equation (7), we considered that daily changes in energy and sodium intake remain constant over time. We assumed that only the warning labels intervention will change the caloric intake, and no other intervention will modify the caloric intake over the five years period that we are simulating.

3.3 Change in BMI

To obtain the expected change in body mass index $BMI_k(t)$ for each individual k, we used Equation (8):

$$BMI_k(t) = BW_k(t)/(H_k)^2, \tag{8}$$

where $BW_k(t)$ represents the estimated individual's body weight (kg) with Hall's model, t stands for the number of days after the intervention, and H_k represents individual's height in meters.

3.4 Change in obesity prevalence

We classified each individual's $BMI_k(t)$ into BMI categories using WHO's cut-off points [14]. We introduced a dummy variable ($BMIcat_k(t)$) defined as:

$$BMIcat_k(t) = \begin{cases} BMIcat_k(t) = 1, & \text{if } BMI_k \geq 30kg/m^2 \\ BMIcat_k(t) = 0, & \text{otherwise,} \end{cases}$$

where $BMIcat_k(t) = 1$ indicates obesity for each individual k in the sample and t stands for the number of days after the intervention.

Then, we calculated the change in obesity prevalence ($\Delta BMIcat_k(t)$) with Equation 9:

$$\Delta BMIcat_k(t) = BMIcat_k(0) - BMIcat_k(t), \tag{9}$$

where k represents each individual in the sample, $BMIcat_k(0)$ corresponds to the baseline BMI category ($t = 0$) and $BMIcat_k(t)$ represents the new BMI category in different time in years.

Finally, since ENSANUT is a cross-sectional, multi-stage, probabilistic survey representative of the Mexican population [15], we used the R package survey [16] to create summary statistics of $BW_k(t)$, $BMI_k(t)$ and $BMIcat_k(t)$ (each in the overall adult population and in specific subpopulations by sex, SES, and age). For these estimates we accounted for the survey design established as follows:

```
svystr <- svydesign(id = ~id , strata = ~est_var , weights =
~pondef , PSU = ~code_upm , data = Adults)
options(survey.lonely.psu = "adjust")
```

4 Obesity cases averted

4.1 Obesity cases

Table K shows the reported obesity prevalence of Mexican adults (20-59 years) in 2012, 2016 and 2018, obtained from the National Health and Nutrition Surveys [1].

Table K: Reported obesity prevalence of Mexican adults (20-59 yrs) from ENSANUT 2012, 2016, 2018.

	2012	2016	2018
Obesity prevalence (%)	34.00%	33.68%	36.77%

To estimate the obesity cases averted, we assumed that obesity prevalence in 2019 was similar to the reported in ENSANUT 2018 and remained constant during the 5 years period. Then, we derived population projections of adults (20 to 59 years) of 2019 to 2023 from the National Council on Population (CONAPO) [17] as seen in Table L:

Table L: Population projections of Mexican adults (20 to 59 yrs) from CONAPO.

	2019	2020	2021	2022	2023
Adult population (20-59)	68,066,782	68,898,173	69,693,988	70,457,557	71,190,536

Lastly, we obtained the total cases of obesity among adults of 20 to 59 years (Table M) as:

$$\text{Obesity cases}_{year} = \text{Adult population}_{year} \times \text{Obesity prevalence}_{2018}, \tag{10}$$

where Adult population_{year} corresponds to the number of people aged 20 to 59 (Table L) at $year = 2019, 2020, ..., 2023$; and Obesity $\text{prevalence}_{2018} = 36.77\%$. The obesity cases from 2019 to 2023, assuming obesity prevalence is in steady state, is presented in the following table.

Table M: Projected obesity cases among Mexican adults.

	2019	2020	2021	2022	2023
Cases of obesity	25,025,229	25,330,896	25,623,483	25,904,214	26,173,699

4.2 Obesity cases averted

To estimate the obesity cases averted, we used the estimated change in obesity prevalence (explained in Section 3). The change in obesity prevalence estimated in the period 2019 to 2023 is presented in Table N.

Table N: Estimated reduction of obesity prevalence in Mexican adults.

	2019	2020	2021	2022	2023
Obesity prevalence reduction (pp)	2.40	3.91	4.67	4.90	4.98

We estimated the total averted cases of obesity for each year (ΔObesity cases$_{year}$) as follows:

$$\Delta\text{Obesity cases}_{year} = \text{Adult population}_{year} \times \Delta\text{Obesity prevalence}_{year}, \quad (11)$$

where Adult population$_{year}$ represents the adult population of 20-59 years at $year = 2019, 2020, ..., 2023$ (Table L); and ΔObesity prevalence$_{year}$ corresponds to the values in Table N. Table O presents the estimated cases averted after implementing the warning labels from 2019 to 2023.

Table O: Estimated reduction of obesity cases among Mexican adults.

	2019	2020	2021	2022	2023
Adverted cases	600,605	990,438	1,196,617	1,269,306	1,303,450

5 Obesity health costs

5.1 Obesity direct and indirect health costs

Direct and indirect health costs of obesity in Mexico were obtained from a report from the Ministry of Health 1999-2023 [18]. This report uses information from two public institutions: Seguro Popular and the Mexican Social Insurance Institute, but does not include out-of-pocket spending. As sources of data, the report includes direct costs from universal health services catalog (CAUSES from its Spanish acronym) from 2014, protection fund for catastrophic expenses (FPGC from its Spanish acronym) from 2014, diagnosis-related group from the Mexican Social Security Institute (GRD-IMSS from its Spanish acronym) from 2012 and diagnosis-related group to Ambulatory Care of Endocrine, Nutritional and Metabolic Diseases (EGRAA from its Spanish acronym) from 2014.
Costs in the report included the overall adult population. To estimate the obesity costs for the target population (20-59 yrs), we first calculated the obesity costs per capita and multiplied it for the disclosed number of adults with obesity (20 to 59 yrs) from the report. Costs are presented in Mexican pesos (MXP). To exemplify (Table P), the direct costs of obesity for the adult population were estimated to be 107.8 billion Mexican pesos (MXP). Divided by the total cases of obesity in that year, direct cost per capita was estimated to be 38,357 pesos. Multiplied by the number of adults with obesity aged 20-59 yrs, we obtained 89.9 billion MXP.

Table P: Direct and indirect costs of obesity among Mexican adults (20 to 59 yrs) in MXP from 2014.

	Total obesity costs (Overall)	Cost/capita	Cases with obesity (20-59 yrs)	Total obesity costs (20-59 yrs)
Direct costs (pesos)	107,796,428,678	38,257	2,350,022	89,904,875,561
Indirect costs (pesos)	72,742,726,794	25,816	2,350,022	60,669,225,137

To obtain the obesity costs (direct and indirect) for the period 2019 to 2023, we first translated costs from 2014 to costs from 2019 (Obesity costs$_{2019}$), dividing total costs from 2014 by the price deflator from the national Consumer Price Index of the National Institute of Statistics and Geography (INEGI from its Spanish acronym) [19]:

$$\text{Obesity costs}_{2019} = \text{Obesity costs}_{2014}/deflator, \quad (12)$$

where Obesity costs_{2014} comes from Table P, column 5; and deflator = 0.82772402. Then we projected the obesity costs from 2019 to 2020, 2021, 2022 and 2023 using a discounted rate of 3% , following standard procedures [20] as in Equation 13:

$$\text{Obesity costs}_{year+1} = \text{Obesity costs}_{year}/(1 + 0.03), \tag{13}$$

where $year = 2019, 2020, 2021, 2022, 2023$; Obesity costs_{year+1} corresponds to the projected health costs of obesity at $year + 1$, and 0.03 corresponds to the 3% discounted rate. Table Q shows the projected direct and indirect costs of obesity in the Mexican adult population (20 to 59 years).

Table Q: Projected direct and indirect health costs (MXP) of obesity in Mexican adults (20 to 59 yrs).

	2019	2020	2021	2022	2023
Direct costs (pesos)	108,616,971,931	105,453,370,807	102,381,913,405	99,399,915,927	96,504,772,745
Indirect costs (pesos)	73,296,442,297	71,161,594,463	69,088,926,663	67,076,627,828	65,122,939,639

5.2 Obesity health costs averted

To calculate the reduction on direct, indirect and total obesity costs in Mexican currency (MXP) (Table R), we used the following equation:

$$\Delta\text{Obesity costs}_{year} = \text{Obesity costs}_{year} \times \text{Obesity prevalence reduction}_{year}, \tag{14}$$

where ΔObesity costs_{year} represents the change in obesity health costs at $year = 2019, 2020,$ ..., 2023; Obesity costs_{year} corresponds to the calculated obesity health costs in Table R and Obesity prevalence reduction_{year} to the estimated obesity prevalence reductions in Table 3.

Table R: Reduction of direct and indirect health costs (MXP) of obesity in Mexican adults (20 to 59 yrs).

	2019	2020	2021	2022	2023	Total (million)
Direct costs (MXP)	2,606,807,326	4,123,226,799	4,781,235,356	4,870,595,880	4,805,937,683	21,188
Indirect costs (MXP)	1,759,114,615	2,782,418,343	3,226,452,875	3,286,754,764	3,243,122,394	14,298
Total costs (MXP)	4,365,921,941	6,905,645,142	8,007,688,231	8,157,350,644	8,049,060,077	35,486

To obtain direct and indirect health costs in US dollars (Table S), we transformed Mexican currency (MXN) to US dollars (USD) using the following relation:

$$1 \text{ MXN from } 2019 = 0.05190017 \text{ USD from } 2019, \tag{15}$$

where the exchange rate used was estimated as the average MXN/USD exchange rate reported by Banxico from January 1st to November 29th of 2019 [21]. Missing data were omitted for the estimation of this rate.

Table S: Reduction of direct and indirect health costs (dollars) of obesity in Mexican adults (20 to 59 yrs).

	2019	2020	2021	2022	2023	Total (million)
Direct costs (dollars)	135,293,743	213,996,172	248,146,928	252,784,754	249,428,983	1100
Indirect costs (dollars)	91,298,348	144,407,985	167,453,453	170,583,131	168,318,604	742
Total costs (dollars)	226,592,091	358,404,157	415,600,381	423,367,885	417,747,586	1842

6 Complementary results

For the convenience of readers, we include the stratified estimates of absolute and relative changes in obesity over 5 years presented in Figure 3 (Table T) and the estimates from the sensitivity analysis presented in Figure 4 (Table U) from the main article.

Table T: Absolute (pp) and percent change (%) of obesity over 5 years by age group, sex and socioeconomic status of the main scenario.

		**Baseline prevalence of obesity (%)**	**2019**	**2020**	**2021**	**2022**	**2023**
Overall		33.8	-2.4 (-7.1)	-3.9 (-11.6)	-4.7 (-13.8)	-4.9 (-14.5)	-4.9 (-14.7)
Age group	20-39	29.3	-2.7 (-9.2)	-4.1 (-13.9)	-4.7 (-16.2)	-5.0 (-17.2)	-5.0 (-17.2)
	40-59	40.1	-1.9 (-4.9)	-3.6 (-9.0)	-4.5 (-11.4)	-4.7 (-11.7)	-4.8 (-12.2)
Sexo	Female	39.0	-2.3 (-6.1)	-4.2 (-10.9)	-5.0 (-12.9)	-5.3 (-13.7)	-5.3 (-13.8)
	Male	27.8	-2.4 (-8.8)	-3.5 (-12.7)	-4.2 (-15.2)	-4.3 (-15.7)	-4.5 (-16.2)
Socioeconomic status	Low	30.1	-1.9 (-6.6)	-2.8 (-9.5)	-3.1 (-10.5)	-3.7 (-12.4)	-3.8 (-12.6)
	Medium	34.8	-3.1 (-9.1)	-4.5 (-13.1)	-5.2 (-14.9)	-5.2 (-15.0)	-5.2 (-15.1)
	High	34.9	-2.1 (-6.0)	-4.0 (-11.5)	-5.0 (-14.5)	-5.2 (-15.1)	-5.4 (-15.5)

Table U: Sensitivity analysis estimates

**Scenarios**	**Main**	**Scenario 1**	**Scenario 2**	**Scenario 3**
**Beverages**				
Definition	Table A		Table B	
Baseline intake (kcal, IC 95%)	220.9 (208.6, 233.2)		295.1 (281.5, 308.7)	
Caloric change (%, IC95%)	-23.2 (-24.5, -21.9)	-22.1 (-23.2, -21.1)	-33.1 (-36.2, -29.9)	-33.1 (-36.4, -29.8)
Body weight (kg, IC 95%)	-1.05 (-1.11, -1.00)	-1.01 (-1.05, -0.96)	-1.46 (-1.6, -1.33)	-1.47 (-1.62, -1.33)
Body Mass Index (kg/m2 IC 95%)	-0.41 (-0.43, -0.38)	-0.39 (-0.41, -0.37)	-0.56 (-0.62, -0.51)	-0.56 (-0.62, -0.51)
Obesity prevalence (pp, IC95%)	-2.92 (-3.67, -0.51)	-3.14 (-3.99, -2.30)	-3.95 (-5.02, -2.88)	-4.08 (-5.11, -3.05)
Cases of obesity (thousand people)	-764	-822	-1034	-1068
**Snacks**				
Definition	Table A		-	-
Baseline intake (kcal, IC 95%)	453.1 (435.5, 470.7)		-	-
Caloric change (%, IC95%)	-13.6 (-14.1, -13.1)	-52.9 (-55.0, -50.9)	-	-
Body weight (kg, IC 95%)	-0.63 (-0.65, -0.60)	-2.41 (-2.50, -2.32)	-	-
Body Mass Index (kg/m2 IC 95%)	-0.25 (-0.26, -0.24)	-0.95 (-0.98, -0.91)	-	-
Obesity prevalence (pp, IC95%)	-1.83 (-2.45, -1.22)	-6.62 (-7.7, -5.55)	-	-
Cases of obesity (thousand people)	-479	-1733	-	-

Main scenario: Effect estimate (-10.5% and -3.0% caloric reduction for beverages and snacks, respectively) based on Canadian experimental study. Scenario 1: Effect estimate (-7.5% caloric reduction) based on Chilean observational study. Scenario 2: Effect estimate (caloric reduction by 27.5% in "high in" beverages and caloric increase by 10-8% in not "high in" beverages) based on Chilean observational study and adding the Chilean limits for the first stage of the law (Table G). Scenario 3: Effect estimate (caloric reduction by 27.5% in "high in" beverages and caloric increase by 10.8% in not "high in" beverages) based on Chilean observational study and adding the Mexican limits (Table I) Scenario 1 Snacks: Effect estimate (-11.68% caloric reduction for snacks) based on Uruguayan study.

7 Assumptions and sources of information to estimate the expected impact of warning labels

In this last section, we present a summary of assumptions to facilitate reading the manuscript.

Assumption	Description	Source
Steady state for beverages and snacks consumption.	We assumed the caloric change attributable to warning labels took place at the beginning of the first year of implementation, remaining constant over time.	ENSANUT 2016 [1].
Caloric change among adults in Mexico would be similar to the estimated caloric change in Canada.	We used an experimental study in Canada to obtain expected changes in calories and sodium .	Acton, et al. [2].
Sodium changes were used only in the Hall's model.	The sodium changes were only only took into consideration for estimating extracellular liquid. Changes in HTA or other diseases were no considered.	Hall, ^{o}et al.,2011 [10].
Steady state condition for obesity prevalence.	We assume that the obesity prevalence is not increasing over time and no changes, in addition to the warning labels, will occur in the 5 years simulation period.	Obesity prevalence to estimate cases of obesity averted was calculated using ENSANUT 2018 (the last available source) [1].
BMI categories	BMI categories for adults were calculated based on WHO cut-offs.	WHO [14].
Obesity costs from 2014	Costs were transformed in 2019 costs using the Mexican inflation index and then discounted 3% per year over 4 years.	Financial report on overweight and obesity in Mexico 1999-2023. National Consumer Price Index from National Institute of Statistics and Geography (INEGI from its Spanish acronym) [18, 19].
	To convert the healthcare costs	

Assumption	Description	Source
Convert Mexican pesos in US Dollars	in Mexican Pesos to US Dollars, we averaged the MXN/USD exchange rates reported by Banxico (FIX rates) in 2019.	Banxico (2019) [21].

References

[1] Instituto Nacional de Salud Pública. Encuesta Nacional de Salud y Nutrición; 2016. Online; accessed 10 November 2019. http://ensanut.insp.mx/.

[2] Acton RB, Jones AC, Kirkpatrick SI, Roberto CA, Hammond D. Taxes and front-of-package labels improve the healthiness of beverage and snack purchases: A randomized experimental marketplace. International Journal of Behavioral Nutrition and Physical Activity. 2019;16:1—-15.

[3] Taillie LS, Reyes M, Colchero MA, Popkin BM, Corvalán C. An evaluation of Chile's Law of Food Labeling and Advertising on sugar-sweetened beverage purchases from 2015 to 2017: A before-and-after study. PLOS Medicine. 2020;17(2):1–22.

[4] R Core Team. R: A Language and Environment for Statistical Computing. Vienna, Austria; 2018. Available from: https://www.R-project.org/.

[5] Government of Canada. Toward Front-of-Package Nutrition Labels for Canadians; 2016. Online; accessed 20 January 2020. https://www.canada.ca/en/health-canada/programs/front-of-package-nutrition-labelling/consultation-document.html#ac2.

[6] Government of Canada. Nutrition Labelling - Table of Reference Amounts for Food; 2016. Online; accessed 20 January 2020. https://www.canada.ca/en/health-canada/services/technical-documents-labelling-requirements/table-reference-amounts-food/nutrition-labelling.html#b.

[7] Corvalán C, Reyes M, Garmendia ML, Uauy R. Structural responses to the obesity and non-communicable diseases epidemic: Update on the Chilean law of food labelling and advertising. Obesity Reviews. 2019;20(3):367–374. Available from: https://onlinelibrary.wiley.com/doi/abs/10.1111/obr.12802.

[8] Romero Anaya R, Novelo Baeza JA. PROYECTO de Modificación a la Norma Oficial Mexicana NOM-051-SCFI/SSA1-2010, Especificaciones generales de etiquetado para alimentos y bebidas no alcohólicas preenvasados-Información comercial y sanitaria, publicada el 5 de abril de 2010;. Diario Oficial de la Federación, Secretaría de Gobernación, México, 2019. https://www.dof.gob.mx/nota_detalle.php?codigo=5575205&fecha=11/10/2019.

[9] Machín L, Curutchet MR, Giménez A, Jessica AW, Gastón A. Do nutritional warnings do their work? Results from a choice experiment involving snack products. Food Quality and Preference. 2019;77:159—-165.

[10] Hall KD, Sacks G, Chandramohan D, Chow CC, Wang YC, Gortmaker SL, et al. Quantification of the effect of energy imbalance on bodyweight. The Lancet. 2011;378(9793):826–837.

[11] Hall KD. Predicting metabolic adaptation, body weight change, and energy intake in humans. American Journal of Physiology-Endocrinology and Metabolism. 2010;298(3):E449–E466.

[12] Hall KD, Jordan PN. Modeling weight-loss maintenance to help prevent body weight regain. The American journal of clinical nutrition. 2008;88(6):1495–1503.

[13] Camacho-García-Formentí D, Zepeda-Tello R. bw: Dynamic Body Weight Models for Children and Adults; 2018. R package version 1.0.0.

[14] World Health Organization. Obesity: preventing and managing the global epidemic. Report of a WHO Consultation.. vol. 894. Geneva: World Health Organization; 2000.

[15] Romero-Martínez M, Shamah-Levy T, Cuevas-Nasu L, Méndez Gómez-Humarán I, Berenice GPE, Gómez-Acosta LM, et al. Diseño metodológico de la Encuesta Nacional de Salud y Nutrición de Medio Camino 2016. Salud Publica de México. 2017;59:1—-299.

[16] Lumley T. survey: analysis of complex survey samples; 2019. R package version 3.35-1.

[17] Consejo Nacional de Población (CONAPO) . Proyecciones de la población de México y de las Entidades Federativas.; 2018. [Online; accessed 28-August-2019]. https://datos.gob.mx/busca/dataset/proyecciones-de-la-poblacion-de-mexico-y-de-las-entidades-federativas-2016-2050.

[18] Secretaria de la Salud. Impacto financiero del Sobrepeso y Obesidad en México 1999-2023. Nota técnica; 2015. Online; accessed 20 November 2019. http://oment.uanl.mx/wp-content/uploads/2016/09/impacto_financiero_OyS_060815_oment.pdf.

[19] Instituto Nacional de Estadística y Geografía. Índice Nacional de Precios al Consumidor (INPC); 2090. Online; accessed 22 November 2019. https://www.inegi.org.mx/temas/inpc/.

[20] Neumann PJ, Sanders GD, Russell LB, Siegel JE, Ganiats TG. Cost-effectiveness in health and medicine. Oxford; New York (NY).

[21] Banco de México. Sistema de información económica. Tipos de cambio diarios.; 2015. Online; accessed 28 November 2019. https://www.banxico.org.mx/SieInternet/consultarDirectorioInternetAction.do?sector=6&accion=consultarCuadro&idCuadro=CF102&locale=es.
