# Supplementary material for: Predicting obesity reduction after implementing warning labels in Mexico: A modeling study
Source: PLoS Med. 2020 Jul 28;17(7):e1003221. doi: 10.1371/journal.pmed.1003221 (PMC7386611; doi:10.1371/journal.pmed.1003221)
Supplement: S1 Parameters — (PDF) [file pmed.1003221.s003.pdf]

Table A S1 Parameters. Model structure and model parameters with their mean values and range when available, sources and caveats.

	Description	Range	Primary source	Caveats
**Model structure**				
	Validated dynamic simulation model of adults, that predicts the time course of individual weight change in response to warning labels in Mexico. We chose this model because it considers dynamic physiological adaptations that occur with body weight changes at individual and population level.	Not applicable	Hall et al [1].	- The model assumes a steady state for each individual's body weight and thus, we could not consider the increase in the obesity trend occurring in Mexico in the last years.<br>- We do not consider potential changes in physical activity over the simulation period and assume a sedentary activity level (1.5) over the simulated period.<br>- A main limitation of the model is how total energy intake is estimated. We refer to this caveat later in the model parameters, below.
**Model parameters**				
Caloric change in beverages	Experimental relative change in caloric consumption of beverages after "high in" warning label in Canadian population	-10.5%	Acton et al [2].	- Experimental study in 3 cities in Canada with adolescents and adults; n = 3584. The 10.5% was only for adults and a sample of 1213 subjects.<br>- Only 20 beverages included (different from the wide range of beverages available in Mexico's marketplace)<br>- The warning label in Canada consists of a single red label with multiple legends and less restrictive thresholds compared to the approved label in Mexico.<br>- Different demographic and socioeconomic characteristics between Canada and Mexico.<br>- The caloric impact could change due to differences in baseline beverage consumption between Mexico and Canada.
	Overall change in caloric consumption of beverages after "high in" experiment in Chilean population<br><br>Change in caloric consumption of "high in" and "not high in" beverages in Chile	Total:<br>-7.5% 95% CI (-7.6%, -7.5%)<br><br>High in:<br>-27.5% 95% CI (-27.6%, -27.5%)<br>Not high in:<br>10.8% 95%	Taille et. al [3].	- The warning label in Chile consists of a black and white stop sign with "high in" for 4 nutrients (similar to Mexico). The proposed nutrient threshold is slightly less restrictive compared to the approved label in Mexico.<br>- The observed caloric changes may include reformulation and consumer behavioral change.<br>- Different population and product availability.

		CI (10.8%, 10.8%)		- Different demographic and socioeconomic characteristics between Chile and Mexico.<br>- Caloric change could be different due to different baseline beverages consumption between Mexico and Chile.
Caloric change in snacks	Experimental relative change in caloric consumption of snacks after "high in" warning label experiment in Canadian population	-3%	Acton et. al [2].	- Experimental study in three cities in Canada with adolescents and adults; n = 3584.<br>- Only 20 snacks Included, in comparison with a wide range of snacks in the Mexican market.
	Experimental relative change in caloric consumption of snacks after "high in" warning label experiment in Uruguayan population	-11.7%	Machin et. al [4].	- The warning label in Uruguay consists of black octagonal signs featuring the expression "Excess" followed by the name of the nutrient.<br>- Experimental study conducted among students and workers from a Montevideo University, Uruguay (n = 199).<br>- Only 15 snacks were included, in comparison with a wide range of snacks in the Mexican market.
Sodium change in beverages	Experimental relative change in sodium consumption of beverages after "high in" warning label in Canadian population	-5.5%	Acton et. al [2].	Idem
Sodium change in snacks	Experimental relative change in sodium consumption of snacks after "high in" warning label in Canadian population	-6.3%	Acton et. al [2].	Idem
	Experimental relative change in sodium consumption of snacks after "high in" warning label in Uruguayan population	-50.2%	Machín et. al [4]	Idem
Baseline total energy intake (kcal)	Total energy intake needed to maintain the baseline bodyweight.	2145.6 95% CI (2130.2, 2161.0)	Mifflin-St Jeor formula for resting energy expenditure [5].<br>Hall et al [1].	- The intervention impact on body weight relies on the baseline total energy intake estimates via Mifflin-St Jeor's validated formula. This formula has been reported as a low precision method to estimate total energy intake, yet widely used because other methods are too expensive [6].

				This uncertainty increases the variability of weight change results even if the expected caloric change is exactly the same for every Mexican adult.<br>- We assume steady state of energy intake throughout time.
Baseline weight (kg)	Individual's measured body weight in kilograms	72.4 95% CI (71.7, 73.0)	ENSANUT 2016 [7].	Results based on estimated population weights.
Baseline height (cm)	Individual's measured baseline height in centimeters	159.7 95% CI (159.3, 160.1)	ENSANUT 2016 [7].	Results based on estimated population weights.
Age	Individual's reported age	37.1 95% CI (36.6, 37.6)	ENSANUT 2016 [7].	Results based on estimated population weights.
Sex	Individual's reported gender	Male 46.6% Female 53.4%	ENSANUT 2016 [7].	Results based on estimated population weights.
Time	Durante of the simulation period.	1-5 years	Study design	Time needed to translate the caloric reduction into weight reductions.
Obesity prevalence	Estimated obesity prevalence in Mexican population	36.77% 95% CI (35.34, 38.19)	ENSANUT 2016 [7].	Results based on estimated population weights.
Population projections of Mexican adults	Projected population of adults (20 to 59 years) in Mexico.	2019 to 2023	National Population Council of Mexico (CONAPO) [8].	Estimates based on observed trends, national census, and validated formulas. Births, deaths and migrations are considered.
Obesity costs in adults 20-59 years	Obesity costs include direct and indirect costs.	Annual expenses of $180.5 billion Mexican pesos in 2014 ($9.4 billion dollars).	Health Ministry [9].	Direct costs include the health system perspective. Out of pocket expenses are not included. However, we included individual perspective in indirect costs.

We included average weight, height, age, obesity prevalence and proportion of males and females, but our model uses individual parameters from the Mexican population.

References

1. Hall KD, Sacks G, Chandramohan D, Chow CC, Wang YC, Gortmaker SL, et al. Quantification of the effect of energy imbalance on bodyweight. Lancet. 2011;378: 826–837. doi:10.1016/S0140-6736(11)60812-X

2. Acton RB, Jones AC, Kirkpatrick SI, Roberto CA, Hammond D. Taxes and front-of-package labels improve the healthiness of beverage and snack purchases: a randomized experimental marketplace. Int J Behav Nutr Phys Act. 2019;16: 46. doi:10.1186/s12966-019-0799-0

3. Taillie LS, Reyes M, Colchero MA, Popkin B, Corvalán C. An evaluation of Chile’s Law of Food Labeling and Advertising on sugar-sweetened beverage purchases from 2015 to 2017: A before-and-after study. PLOS Med. 2020;17: e1003015. Available: https://doi.org/10.1371/journal.pmed.1003015

4. Machín L, Curutchet MR, Giménez A, Aschemann-Witzel J, Ares G. Do nutritional warnings do their work? Results from a choice experiment involving snack products. Food Qual Prefer. 2019;77: 159–165. doi:https://doi.org/10.1016/j.foodqual.2019.05.012

5. Mifflin MD, St Jeor ST, Hill LA, Scott BJ, Daugherty SA, Koh YO. A new predictive equation for resting energy expenditure in healthy individuals. Am J Clin Nutr. 1990;51: 241–247. doi:10.1093/ajcn/51.2.241

6. Schoeller DA, Fjeld CR. Human energy metabolism: what have we learned from the doubly labeled water method? Annu Rev Nutr. 1991;11: 355–373. doi:10.1146/annurev.nu.11.070191.002035

7. Romero-Martínez M and et al. Diseño metodológico de la Encuesta Nacional de Salud y Nutrición de Medio Camino 2016. Salud Publica Mex. 2017;59: 299–305. doi:http://dx.doi.org/10.21149/8593.

8. (CONAPO) CN de P. Proyecciones de la población de México y de las Entidades Federativas. 2018.

9. Secretaría de Salud G de M. Impacto financiero del Sobrepeso y Obesidad en México 1999-2023. Nota técnica. 2015 [cited 20 Nov 2019]. Available: http://oment.uanl.mx/wp-%0A content/uploads/2016/09/impacto_financiero_OyS_060815_oment.pdf
